# Supplementary figures and images for: Experimental H1N1pdm09 infection in pigs mimics human seasonal influenza infections
Source: PLoS One. 2019 Sep 20;14(9):e0222943. doi: 10.1371/journal.pone.0222943 (PMC6754157; doi:10.1371/journal.pone.0222943)

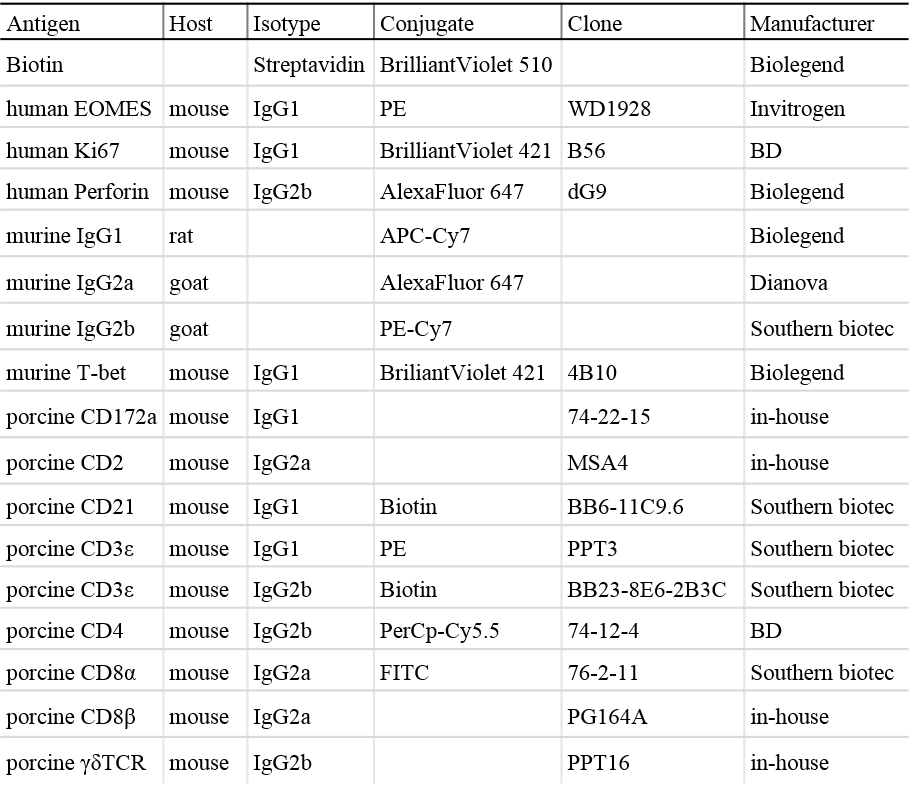

Supplement: S1 Table — (TIF) [file pone.0222943.s001.tif]
